# Supporting Ontario public health units to address adverse childhood experiences in pandemic recovery planning: A priority-setting exercise

**DOI:** 10.1186/s12961-024-01156-0

**Published:** 2024-06-13

**Authors:** Kimberly B. Harding, Erica Di Ruggiero, Erick Gonzalez, Amanda Hicks, Daniel W. Harrington, Sarah Carsley

**Affiliations:** 1 Dalla Lana School of Public Health, University of Toronto, 155 College St, Toronto, ON M5T 3M7 Canada; 2 Family Health Division, Niagara Region Public Health and Emergency Services, 1815 Sir Isaac Brock Way, Thorold, ON L2V 4Y6 Canada; 3 School of Nursing, McMaster University, 1280 Main Street West, Hamilton, ON L8S 4L8 Canada; 4 Health Promotion, Chronic Disease and Injury Prevention, Public Health Ontario, 661 University Avenue, Suite 1701, Toronto, ON M5G 1M1 Canada

**Keywords:** Adverse childhood experiences, COVID-19 pandemic recovery, priority-setting, Public health, Maternal and child health, Healthy growth and development

## Abstract

**Background:**

Adverse childhood experiences (ACEs) are potentially traumatic exposures experienced during childhood, for example, neglect. There is growing evidence that the coronavirus disease 2019 (COVID-19) pandemic and related socioeconomic conditions contributed to an increased risk of ACEs. As public health programs/services are re-evaluated and restored following the state of emergency, it is important to plan using an ACEs-informed lens. The aim of this study was to identify and prioritize initiatives or activities that Public Health Ontario (PHO) could undertake to support Ontario public health units’ work towards ACEs-informed pandemic recovery plans.

**Methods:**

The Child Health and Nutrition Research Initiative method was adapted to conduct a priority-setting exercise (May–October 2022). Two online surveys were administered with members of the Healthy Growth and Development (HGD) Evidence Network, comprised of public health unit staff working in child and family health/HGD from Ontario’s 34 public health units. In the first survey, participants were asked to propose activities or initiatives that PHO could undertake to support Ontario public health units’ work towards ACEs-informed planning. In the second survey, participants were asked to score the final list of options against pre-determined prioritization criteria (for example, relevance). Responses were numerically coded and used to calculate prioritization scores, which were used to rank the options.

**Results:**

In all, 76% of public health units (*n* = 26) responded to the first survey to identify options. The 168 proposed ideas were consolidated into a final list of 13 options, which fall under PHO’s scientific and technical support mandate areas (data and surveillance, evidence synthesis, collaboration and networking, knowledge exchange and research). A total of 79% of public health units (*n* = 27) responded to the follow-up survey to prioritize options. Prioritization scores ranged from 76.4% to 88.6%. The top-ranked option was the establishment of a new provincial ACEs community of practice.

**Conclusions:**

Over three quarters of public health units contributed to identifying and ranking 13 options for PHO to support public health units in considering and addressing ACEs through pandemic recovery planning. In consultation with the ACEs and Resilience Community of Practice, recently formed on the basis of this exercise, PHO will continue to use the ranked list of options to inform work-planning activities/priorities.

## Background

The coronavirus disease 2019 (COVID-19) pandemic posed unprecedented challenges to public health, disrupting communities, economies and the overall well-being of individuals and populations globally. In the wake of this crisis, it has become evident that the effects of the pandemic and the emergency measures used to mitigate disease transmission extended well beyond the immediate health implications of the virus. In Canada, lockdown periods and school closures were the second longest in the world at 51 weeks [1]. The financial impact, increased household stress and disruption of support systems contributed to an environment that increased the risk of adverse childhood experiences (ACEs), including child maltreatment and household dysfunction [2–4]. There is also evidence that poor mental health outcomes increased during the pandemic, particularly among young girls and women [5]. As public health practitioners are transitioning back from the state of emergency and preparing to address these emerging public health challenges, it will be important to plan public health programs with an ACE-informed lens.

In Ontario, 34 public health units are responsible for delivering public health programs and services, as per the Ontario Public Health Standards [6]. One of the program standards is Healthy Growth and Development (HGD), which is aimed at achieving “optimal preconception, pregnancy, newborn, child, youth, parental, and family health” [7]. One area of focus for HGD is ACEs, which are “potentially traumatic exposures that individuals may experience during childhood ages 0 to 18 years” such as physical or emotional abuse, neglect, household dysfunction or exposure to violence [8]. These experiences can have profound and lasting effects on a child’s physical, mental and social development, leading to a wide range of negative health outcomes in adulthood [9, 10]. ACEs have been linked to increased risks of chronic diseases, mental health and substance use disorders and poor health behaviours [11]. Addressing ACEs has been established as a public health priority by Ontario’s public health units [12, 13].

Public Health Ontario (PHO) is a provincial Crown Agency with a mandate to “provide scientific and technical advice and support to clients working in government, public health, health care, and related sectors” [14]. These clients include Ontario’s 34 public health units. As part of this support, and in response to a previous needs assessment [15], PHO convenes the HGD Evidence Network to enhance province-wide collaboration and share innovative research, evidence synthesis and best practices to advance evidence-based public health practice that supports the early years, healthy communities and reducing health inequities. Membership includes public health staff in multiple positions including senior leadership (for example, directors and managers) and frontline staff (for example, public health nurses and health promoters) working in family health and HGD from the 34 public health units. The HGD Evidence Network meets virtually bi-monthly. Through this network, PHO can plan and assess the evidence needs of public health practitioners providing services to children and families across Ontario, including supporting COVID recovery efforts. Once the COVID-19 pandemic response moved towards the recovery stage, there was strong interest among the HGD Evidence Network in return-to-work planning.

The social, economic and psychological consequences of the pandemic disproportionately impacted underserved populations, including children who have experienced ACEs and their families [16]. Therefore, pandemic recovery efforts should be informed by trauma-informed approaches, which emphasize understanding and addressing the impact of trauma and of ACEs and their potential long-term effects. The aim of this study was to identify and prioritize initiatives or activities that PHO could undertake to support Ontario public health units’ work towards ACEs-informed recovery plans.

## Methods

**Fig. 1:**
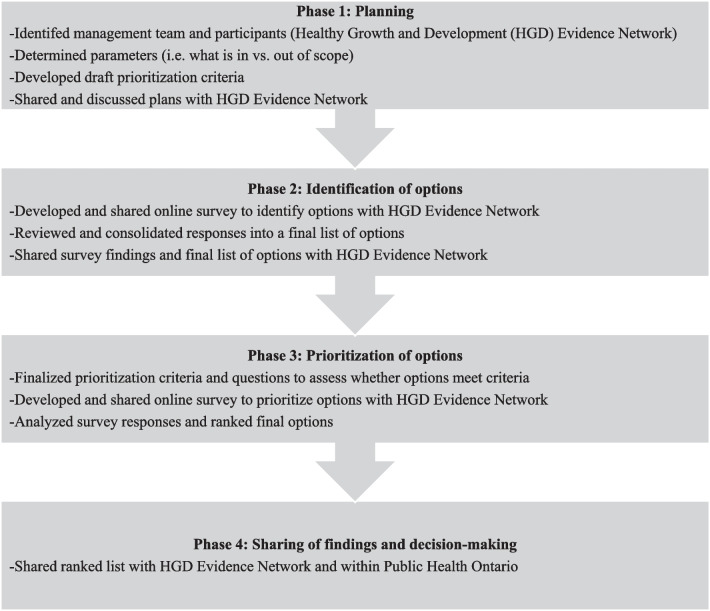
Phases and steps followed for the priority-setting exercise

We followed the Child Health and Nutrition Research Initiative (CHNRI) method, a widely used, participatory and adaptable approach for setting health research priorities [17, 18]. Though initially designed and typically used for identifying research priorities [18], the method has also been applied to other contexts and needs [19]. We adapted the recommended CHNRI method into four phases, each containing multiple steps (Fig. 1) and conducted the priority-setting exercise between May and October 2022. The priority-setting exercise was approved by the PHO Ethics Review Board, and we obtained informed consent from all participants.

### Phase 1: Planning (May–June 2022)

In the planning phase, a core management team responsible for designing and implementing the process (KBH and SC) was identified, along with guidance from advisors consulted throughout (EDR, EG, EH and DH). In addition, participants for the priority-setting exercise were identified as public health unit staff working in child and family health/HGD from Ontario’s 34 public health units who participate in the HGD Evidence Network.

We developed parameters for the priority-setting exercise, which outlined what was in and out of scope (Table 1). An initial list of prioritization criteria, informed by those recommended for the CHNRI method and used in previous CHNRI exercises [17, 18], was also developed and later finalized in Phase 3. In the planning phase, plans for the priority-setting exercise were also shared and discussed with the HGD Evidence Network members during a regular meeting.

**Table 1:** Parameters shared with participants for the priority-setting exercise

In scope	Out of scope
Initiatives or activities that would: Support Ontario public health units’ work towards ACEs-informed recovery plans Fall under Public Health Ontario (PHO)’s scientific and technical support mandate (specifically, data and surveillance, evidence synthesis, collaboration and networking, knowledge exchange and research) Be undertaken as a collaboration between public health units and PHO, or solely by PHO	Anything related to: Policy and strategic direction for the province of Ontario Financial support Providing ACEs or trauma-informed training or capacity-building opportunities

### Phase 2: Identification of options (July–August 2022)

To develop a list of options for prioritization, we created an online survey using the PHO survey platform and invited by email HGD Evidence Network members to participate (*N* = 91 members from 34 public health units). Respondents were reminded of the parameters and asked what activities or initiatives PHO could undertake to support Ontario public health units’ work towards ACEs-informed recovery plans. They were also asked how strong the focus on ACEs was in their public health unit’s pandemic recovery planning so far. All 34 public health units were represented in the HGD Evidence Network, and one response per public health unit was requested. Respondents were encouraged to consult with relevant colleagues within their public health unit for their responses. In addition to the HGD Evidence Network members, the PHO Applied Public Health Science Specialist in Healthy Growth and Development (Chair of the HGD Evidence Network and member of the core management team for this exercise) also contributed ideas to the compiled list of responses.

If more than one response was received from a public health unit, all proposed ideas were included in the initial list. We reviewed and consolidated the initial list of proposed ideas into a final list of options. This involved removing those which were out of scope based on the pre-specified parameters and those which provided insufficient information. The remaining potential options were then coded on the basis of the five key areas of PHO’s scientific and technical support mandate that formed part of the initial parameters (data and surveillance, evidence synthesis, collaboration and networking, knowledge exchange and research). Duplicate suggestions were removed, and similar ideas were consolidated. To limit the number of final options, only those ideas proposed at least twice by respondents were included in the final list. We shared findings from the first survey, including the final list of options, with the HGD Evidence Network members during a regular meeting.

### Phase 3: Prioritization of options (August–September 2022)

In the prioritization phase, we finalized the prioritization criteria and created agreement statements to assess the extent to which, from the perspective of the respondents, each of the final options met each of the three criteria (Table 2). A limited set of prioritization criteria were chosen for simplicity and to help public health units identify what would be most beneficial to their practice. To prioritize the final list of options, we developed a second online survey and emailed an invitation to participate with the survey link to the HGD Evidence Network members. For each of the final options, respondents were asked to respond to agreement statements for each of the three prioritization criteria using the following Likert scale response options: strongly agree, agree, neither agree nor disagree, disagree or strongly disagree. One response per public health unit was requested. Public health units who did not participate in Phase 2 were still invited to participate in Phase 3.

**Table 2:** Prioritization criteria and agreement statements for the priority-setting exercise

Prioritization criterion	Agreement statement
Relevance	This initiative/activity is relevant to the recovery work we are planning in our health unit.
Need	This initiative/activity is needed to advance the recovery work we are planning in our health unit.
Impact	This initiative/activity would help my health unit contribute to improving health and equity in our population.

To analyse the results, we numerically coded the participant responses to the agreement statements (strongly agree = 1, agree = 0.75, neither agree nor disagree = 0.5, disagree = 0.25 and strongly disagree = 0). If more than one response was received for a public health unit, all complete responses were averaged after coding to create one value per public health unit for each agreement statement. Then, for each option, a criterion score for each of the three prioritization criteria was created by averaging values for all of the public health unit’s responses to each agreement statement. For each option, the three criterion scores were then averaged to create an overall prioritization score. The prioritization scores were then used to rank the options. The Pearson correlation coefficient was also calculated to quantify the association between the number of times an option was proposed and its prioritization score. In addition, for each option we calculated the average expert agreement, which reflects the average proportion of respondents who submitted the most common response (that is, the mode) for each of the three agreement statements. All data analysis was conducted using Microsoft Excel (v 16.66.1).

### Phase 4: Sharing of findings and decision-making (September–October 2022)

We shared the ranked list of options, based on the prioritization survey results, with HGD Evidence Network members during a regular meeting held in October 2022, which included a discussion of next steps to action the top prioritized areas of work. The findings were also presented to the Department of Health Promotion, Chronic Disease and Injury Prevention at PHO during a departmental meeting in September 2022. Subsequently, the list was used by management and technical staff within the Department of Health Promotion, Chronic Disease and Injury Prevention to inform work-planning decisions.

## Results

We received responses to the identification survey from 26 of 34 (76%) public health units and from 27 (79%) public health units for the prioritization survey. In the prioritization survey, most (*n* = 25) submitted one response. Two public health units submitted more than one response (one submitted two, and one submitted three responses), and their responses were averaged to obtain one score per public health unit. Two health units did not respond to either the identification or the prioritization surveys.

### Identification of options

Over one third of respondents reported that their public health unit had either a strong or very strong focus on ACEs as part of pandemic recovery planning (7.7% and 30.8%, respectively). Approximately half reported some or minor consideration of ACEs (42.3% and 15.4%, respectively). One (3.8%) reported no consideration of ACEs at all in recovery planning.

**Fig. 2:**
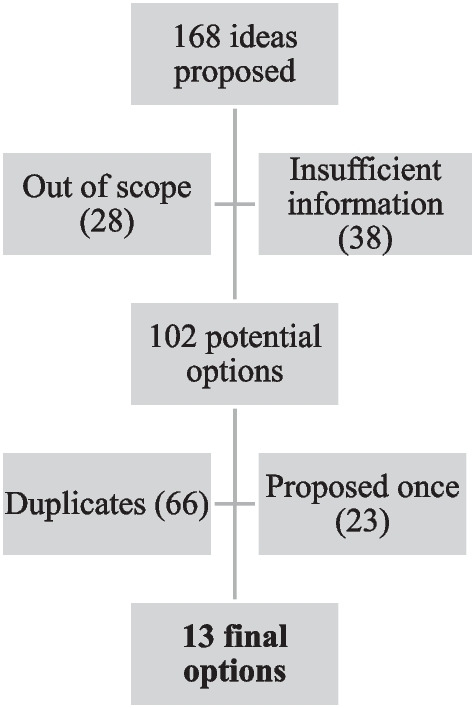
Flow chart of process to review/consolidate the proposed ideas into a final list of options

The respondents proposed a total of 168 ideas, 66 of which were removed because they were out of scope (*n* = 28) or had insufficient information (*n* = 38; Fig. 2). After consolidating similar options and removing duplicates (*n* = 66) as well as those that were only proposed once (*n* = 23), the final list included 13 options (Table 3).

**Table 3:** Final options for Public Health Ontario to support public health units with ACEs-informed pandemic recovery planning

Theme	Proposed option	Number of respondents that proposed the option
Data and surveillance	Design and obtain ethical approval for a standardized provincial ACEs survey for public health units to implement, and provide support to public health units for data analysis and reporting	7
	Develop an ACEs recovery dashboard with guidance on what indicators should be monitored during the recovery period, including risk and protective factors	4
	Develop guidance on ACEs data, including information on existing and new data sources, indicators and how to use/report data	9
Evidence synthesis	Literature review on the “data story” of how ACEs and risk and protective factors were impacted during the pandemic	2
	Literature review on promising practices for increasing awareness of ACEs among different target audiences	2
	Literature review on promising practices for fostering collaborations to address ACEs with different types of partners	5
	Literature review on implementation factors associated with success/impact of public health programs to address ACEs	2
Collaboration and networking	Create a new provincial ACEs community of practice to support public health units to work on common goals and activities, co-chaired by PHO and public health unit representative(s)	19
	Facilitate collaboration with the Association of Public Health Epidemiologists in Ontario, for example, to explore whether ACEs indicators could be added to their Core Indicators Table	2
Knowledge exchange	Develop a toolkit of ACEs knowledge exchange resources for public health units to use/adapt (would involve a mapping exercise to identify gaps and then potentially developing resources where needed)	15
	Develop guidance on how to incorporate an ACEs lens into recovery planning	4
	Host PHO events to create awareness about ACEs among different target audiences (for example, Grand Rounds, fireside chats)	2
Research	Support research on ACEs and the Healthy Babies Healthy Children program, using program data, and facilitate collaborations with academics and other stakeholders	3

ACEs, adverse childhood experiences; PHO, Public Health Ontario

### Prioritization of options.

Prioritization scores ranged from 76.4% to 88.6% (Table 4). All five themes appeared within the top six ranked options. The correlation between the prioritization score and the number of times an option was proposed was *r* = 0.55 (*P* = 0.05). The top two ranked options were also the most frequently proposed; a community of practice was proposed 19 times, and toolkit of knowledge exchange resources was proposed 15 times (Table 3). Average expert agreement ranged from 39.5% to 56.8%.

**Table 4:** Ranked options for Public Health Ontario to support public health units with ACEs-informed pandemic recovery planning

Rank	Summary of option	Theme	Prioritization score (%)	Criterion score (%)			Average expert agreement (%)
				Relevance	Need	Impact	
1	New provincial ACEs community of practice	Collaboration and networking	88.6	91.4	86.3	88.1	50.6
2	Toolkit of ACEs knowledge exchange resources	Knowledge exchange	87.2	89.0	84.4	88.1	48.1
3	Literature review on increasing awareness of ACEs	Evidence synthesis	86.0	90.4	79.5	88.1	53.1
4	Guidance on ACEs data	Data and surveillance	85.8	87.2	83.0	87.2	51.9
5	ACEs recovery dashboard	Data and surveillance	84.6	86.4	81.6	85.6	55.6
6	Research on ACEs and the Healthy Babies Healthy Children program	Research	84.0	86.3	81.3	84.4	39.5
7	Literature review on factors associated with the ACEs program success	Evidence synthesis	83.8	86.3	79.9	85.3	51.9
8	Literature review on ACEs pandemic “data story”	Evidence synthesis	83.8	86.6	79.6	85.2	56.8
9	Guidance on ACEs and recovery planning	Knowledge exchange	83.2	85.2	79.2	85.2	44.4
10	Collaboration with Association of Public Health Epidemiologists in Ontario	Collaboration and networking	82.2	85.0	76.1	85.5	51.9
11	Provincial ACEs survey	Data and surveillance	81.3	86.1	75.2	82.6	49.4
12	Host PHO events to create awareness about ACEs	Knowledge exchange	78.5	81.2	76.5	77.9	39.5
13	Literature review on fostering ACEs collaborations	Evidence synthesis	76.4	78.7	72.7	77.9	46.9

ACEs, adverse childhood experiences; PHO, Public Health Ontario

## Discussion

In this priority-setting exercise, members of the HGD Evidence Network, representing over three quarters of Ontario public health units, contributed to the identification and ranking of 13 options for initiatives or activities that PHO could undertake to support public health units with ACEs-informed pandemic recovery planning. These options fall under PHO’s scientific and technical support mandate areas of data and surveillance, evidence synthesis, collaboration and networking, knowledge exchange and research.

The top-ranked option was the creation of a new provincial ACEs community of practice for supporting public health units to work on common goals and activities. Prior to the COVID-19 pandemic, PHO convened an ACEs Collaborative Working Group with a subset of public health units for two projects, a literature review on public health approaches implemented in Canada for preventing and mitigating the impact of ACEs [12] and an environmental scan of public health programs in Ontario to address ACEs [13]. The ACEs Collaborative Working Group demonstrated a successful partnership between PHO and public health units; however; it was disbanded in March 2020 due to public health unit staff redeployment to the COVID-19 emergency response. In addition to having the highest prioritization score, this option was also the most frequently proposed (19 times, representing 73% of responding public health units), demonstrating a high demand for peer collaboration and networking. A new ACEs and Resilience Community of Practice has since been convened by PHO, based on this exercise. In January 2023, the ACEs and Resilience Community of Practice held its first meeting. It is co-chaired by PHO and a manager from a public health unit and meets monthly. In the first 6 months, meeting attendance has grown to between 50–60 members representing on average 25 public health units. The overarching objective of the community of practice is to foster collaboration and networking to enhance public health-related ACEs and resilience initiatives across Ontario through facilitating knowledge exchange, best practices and evidence-based interventions. This sharing and learning from others may also help to maximize public health unit resources, by reducing the work that is done independently by individual public health units.

The second-ranked option was the development of a toolkit of ACEs knowledge exchange resources for public health units to use or adapt. In addition to having the second-highest prioritization score, this was also the second most frequently proposed option (mentioned 15 times, representing 58% of the responding public health units). Niagara Region Public Health and Emergency Services has undertaken a knowledge translation project to increase awareness of ACEs among internal and external partners, which has involved the development of knowledge translation products and delivery approaches – for example, a “Fostering resilience in Niagara” workshop and self-directed learning package and physician newsletter inserts. This work was previously shared with the HGD Evidence Network, which may have influenced the proposed ideas and also the scoring of this option.

PHO in consultation with the ACEs and Resilience Community of Practice will continue to use the ranked list of options to inform work-planning decisions. The process developed for this exercise has also been used for priority-setting with public health unit partners by other PHO content areas, including Injury Prevention, School Health, and Healthy Eating and Food Environments.

Though not specific to public health and the COVID pandemic or recovery, there are some similarities between our findings and other ACEs priority-setting exercises. For example, one short-term research, policy and practice opportunity identified to address ACEs through pediatrics and children’s health services across the United States was linking with collaborative learning and research networks [20]. This is similar to the ACEs community of practice option under the collaboration and networking theme in our exercise. In addition, through an initiative to build a trauma-informed and resilient community in Pennsylvania, community stakeholders identified communication and networking as key areas for action [21]. Although this work was described as a planning rather than a priority-setting exercise, the participatory process followed ultimately represents community priorities. It is also similar to both the knowledge exchange and the collaboration and networking themes from our exercise.

Average expert agreement was low compared with other CHNRI exercises. This may be due to averaging the agreement statement response values when multiple responses were received from the same health unit, which lowered the average expert agreement, as it created several unique values (outside of the standard 0, 0.25, 0.5, 0.75, and 1). Another reason why this study found lower expert agreement might be the diverse geography and populations across the most populous province in Ontario, Canada. The relevance, need and impact of each option would be dependent on each public health units’ local context. Another group which similarly used the CHNRI method for a topic that did not have a research focus also reported low agreement, though it was slightly higher than in this study (0.48–0.73) [19].

There are several strengths to our approach. This priority-setting exercise followed a systematic, transparent and participatory approach, based on an established method that involves scoring options against pre-determined criteria to create a ranked list. The participatory process built on an existing network with established relationships, and the topic of the priority-setting exercise was determined on the basis of interests and needs previously identified by this group. Both factors may have contributed to the relatively high level of engagement seen in the surveys. The response rates were slightly higher than the average for online surveys with a sample size of 100 or fewer, which has been estimated at 73% [22]. We considered the response rates especially good in the context of pandemic recovery. In addition, the core team and advisor members from PHO were in a position to act on the basis of the findings, which meant that the ranked list was immediately used to inform decision-making. This exercise adds to the limited body of evidence demonstrating the potential use of the CHNRI method for applied public health topics beyond research.

There were also limitations to this work. Though all Ontario public health units were invited to participate in the priority-setting exercise, not all responded to the surveys. Eight (24%) and seven (21%) public health units did not respond to the identification and prioritization surveys, respectively. This may have affected the ideas proposed, how the final list of options was ranked and the overall generalizability to all of Ontario. However, there did not appear to be a pattern for non-respondents in terms of geographical location within the province (there were only two public health units that did not participate in either of the two surveys). The consolidation of the 102 potential options down to the final list of 13 options resulted in the removal of any idea proposed only once but was necessary to improve clarity and ensure the length of the prioritization survey was feasible to complete. The full list was not shared back with the public health units but is available for reference as needed. However, this may have eliminated options that would have possibly ranked high. Though the prioritization criteria were shared with the HGD Evidence Network in advance, these stakeholders (beyond the core group and advisors) were not involved in the development of the criteria. The ranking may have been different had other criteria been selected. Another possible limitation is that some modifications were made to the CHNRI method for the purpose of this exercise. Although adaptations are recommended and often used [17, 18], this may have affected our findings. For example, it is recommended that multiple yes/no questions are developed to determine whether proposed options satisfy each criterion [17]. We used a single agreement statement for each criterion with Likert scale response options to simplify the survey and better capture variation in responses. It is also recommended that additional non-technical stakeholders provide input to the prioritization criteria, including developing criterion thresholds and weights [18]. No thresholds or weights were used for the criteria in this exercise. Finally, although this prioritization exercise provides a rigorous method to support the needs of public health, it was specific to a moment in time in the context of pandemic recovery, and priorities may change. Moving forward, through the ACEs and Resilience Community of Practice and the HGD Evidence Network, all public health units will have the opportunity to shape, re-shape and contribute to the direction and implementation of projects taken on by PHO.

## Conclusions

A majority of public health units in Ontario contributed to identifying and ranking 13 initiatives or activities that PHO could undertake to support addressing ACEs throughout pandemic recovery planning. As a result of this exercise, the ACEs and Resilience Community of Practice was formed. This community of practice has enabled public health units to work collaboratively among themselves as well as with PHO. As the full impact of the COVID-19 pandemic is studied and public health units and the health system continue to recover, the work identified and prioritized by this study aims to support communities and public health units to improve important health outcomes. Maintaining consistent communication and partnership with public health units allows PHO to be proactive in addressing the needs of their main clients whilst supporting standardization and consistency across the province where applicable. PHO will continue to use the ranked list of options to inform work-planning decisions in partnership with the public health units.

## Data Availability

The dataset used and/or analysed during the current study is available from the corresponding author on reasonable request.

## Abbreviations

ACEs: Adverse childhood experiences
CHNRI: Children Health and Nutrition Research Initiative
HGD: Healthy Growth and Development
PHO: Public Health Ontario
